# Supplementary material for: Global land use changes are four times greater than previously estimated
Source: Nat Commun. 2021 May 11;12:2501. doi: 10.1038/s41467-021-22702-2 (PMC8113269; doi:10.1038/s41467-021-22702-2)
Supplement: Supplementary file 1 — Supplementary Information [file 41467_2021_22702_MOESM1_ESM.pdf]

Supplementary information for

"Global land use changes are four times greater than previously estimated"

Karina Winkler[1,2], Richard Fuchs[2], Mark Rounsevell[2,3,4], and Martin Herold[1]

[1] Laboratory of Geoinformation and Remote Sensing, Wageningen University & Research (WUR), The Netherlands
[2] Land Use Change & Climate Research Group, IMK-IFU, Karlsruhe Institute of Technology (KIT), Germany
[3] Institute of Geography & Geo-ecology (IFGG), Karlsruhe Institute of Technology, Germany
[4] School of Geosciences, University of Edinburgh, UK

Content

Supplementary Figures

Supplementary Figure 1: Production of different crop types.. 3
Supplementary Figure 2: Agricultural exports by value (billion US$) 4
Supplementary Figure 3: Annual rate of land use change by agro-ecological zones. 5
Supplementary Figure 4: Annual rate of land use change for countries and world regions... 6
Supplementary Figure 5: Annual rates of land use change related to agriculture................ 7
Supplementary Figure 6: Spatial distribution of global mean uncertainty for HILDA+....... 8
Supplementary Figure 7: Spatial distribution of quality flags for HILDA+........................10
Supplementary Figure 8: Graphical overview of the HILDA+ (HIstoric Land Dynamics Assessment+) framework 11
Supplementary Figure 9: Methodological steps of the land use/cover (LUC) change allocation procedure 12

Supplementary Tables

Supplementary Table 1: Land use/cover (LUC) datasets used for HiLDA+.......................13
Supplementary Table 2: Defined target land use/cover categories of HILDA+ 15
Supplementary Table 3: Generalised land cover classes used for dataset harmonisation....... 5
Supplementary Table 4: Quality flags for HILDA+. 6
Supplementary Table 5: Rules for assembling class probability maps.................................. 6
Supplementary Table 6: Rule-set for calibrating the base map to FAO land use 7
Supplementary Table 7: Countries with changes in area in 1960-2015................................. 8

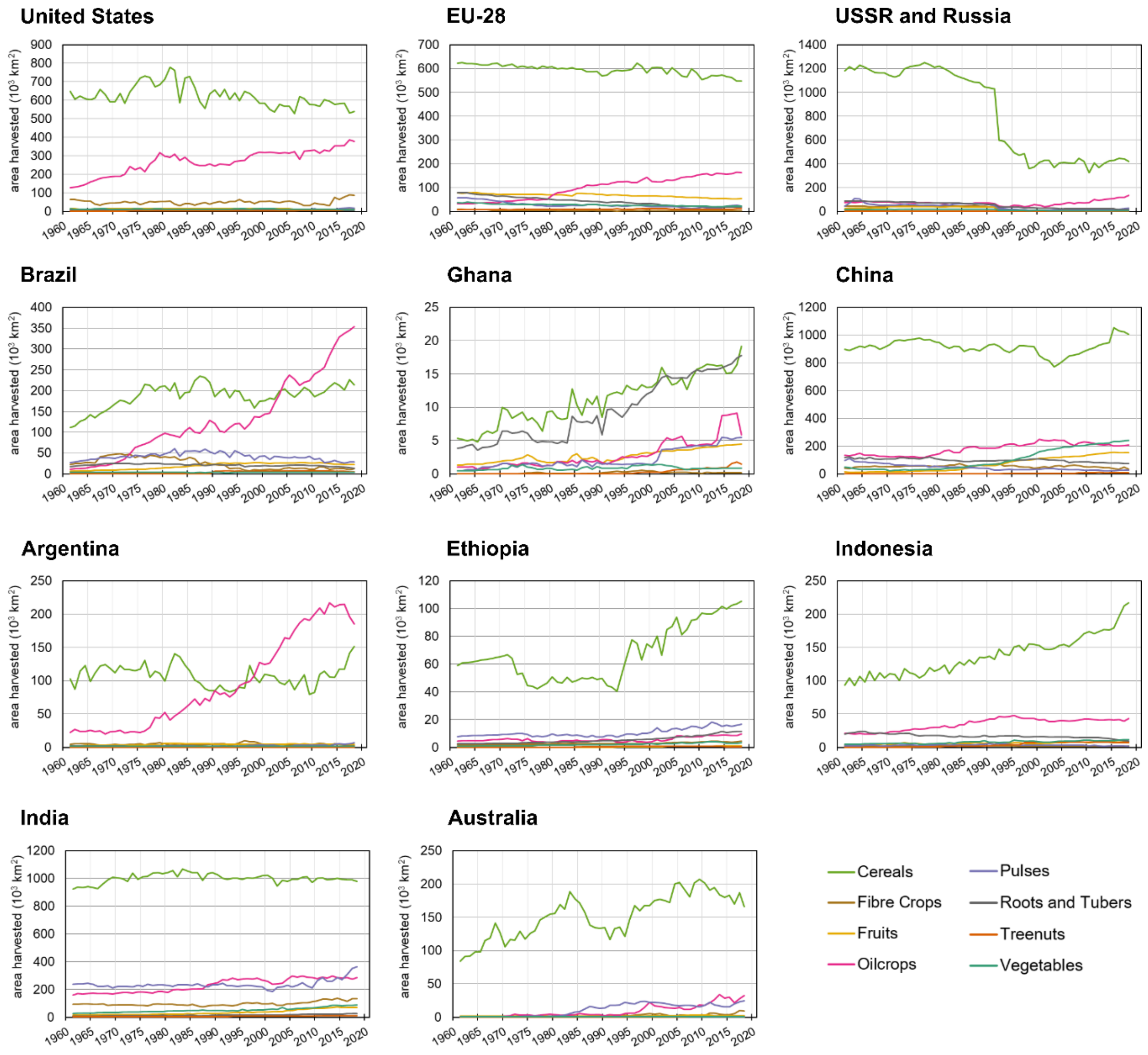

Supplementary Figure 1: Production of different crop types by area harvested for different countries and world region[25]

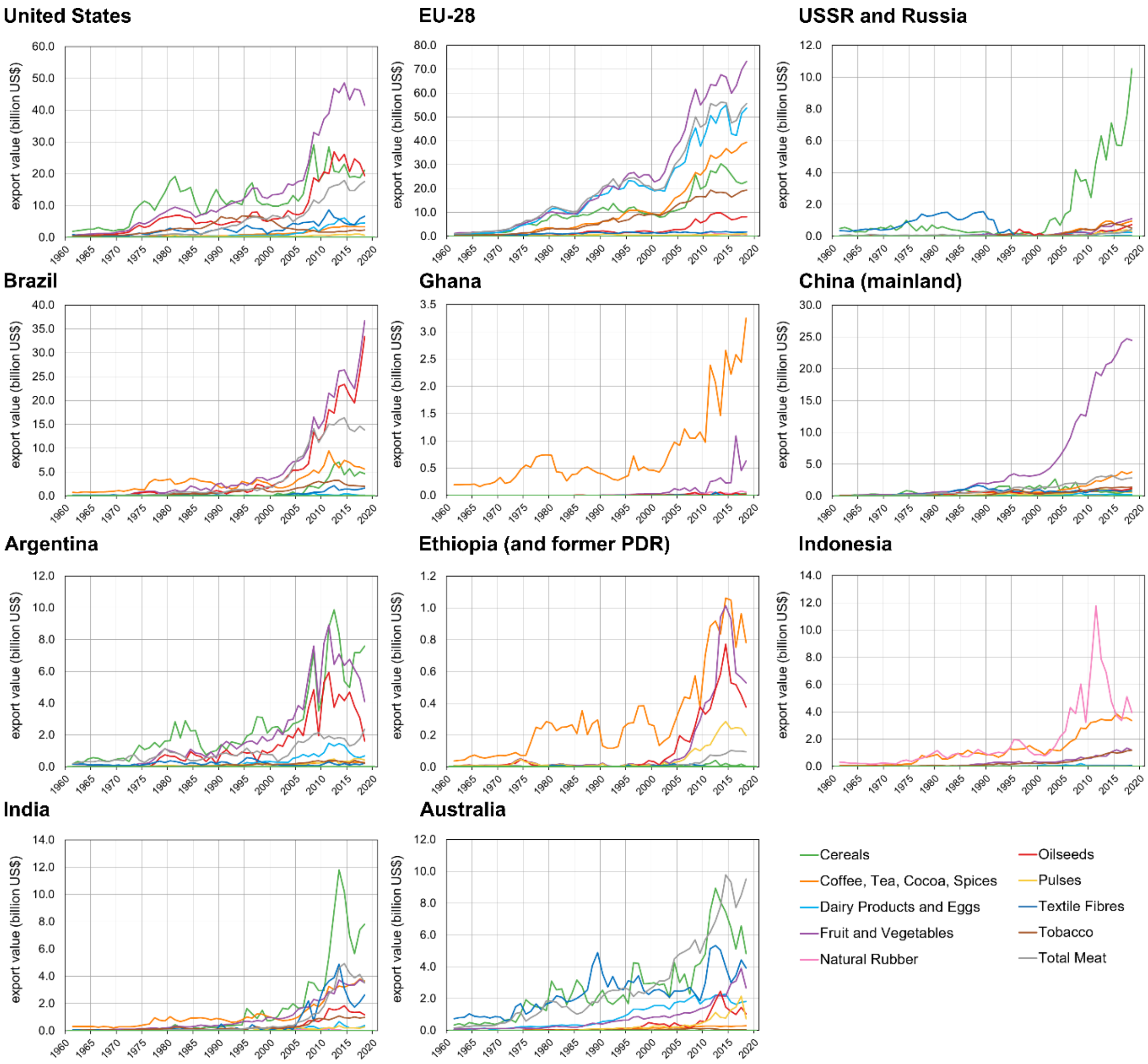

Supplementary Figure 2: Agricultural exports by value (billion US$) for different countries and world regions[24]

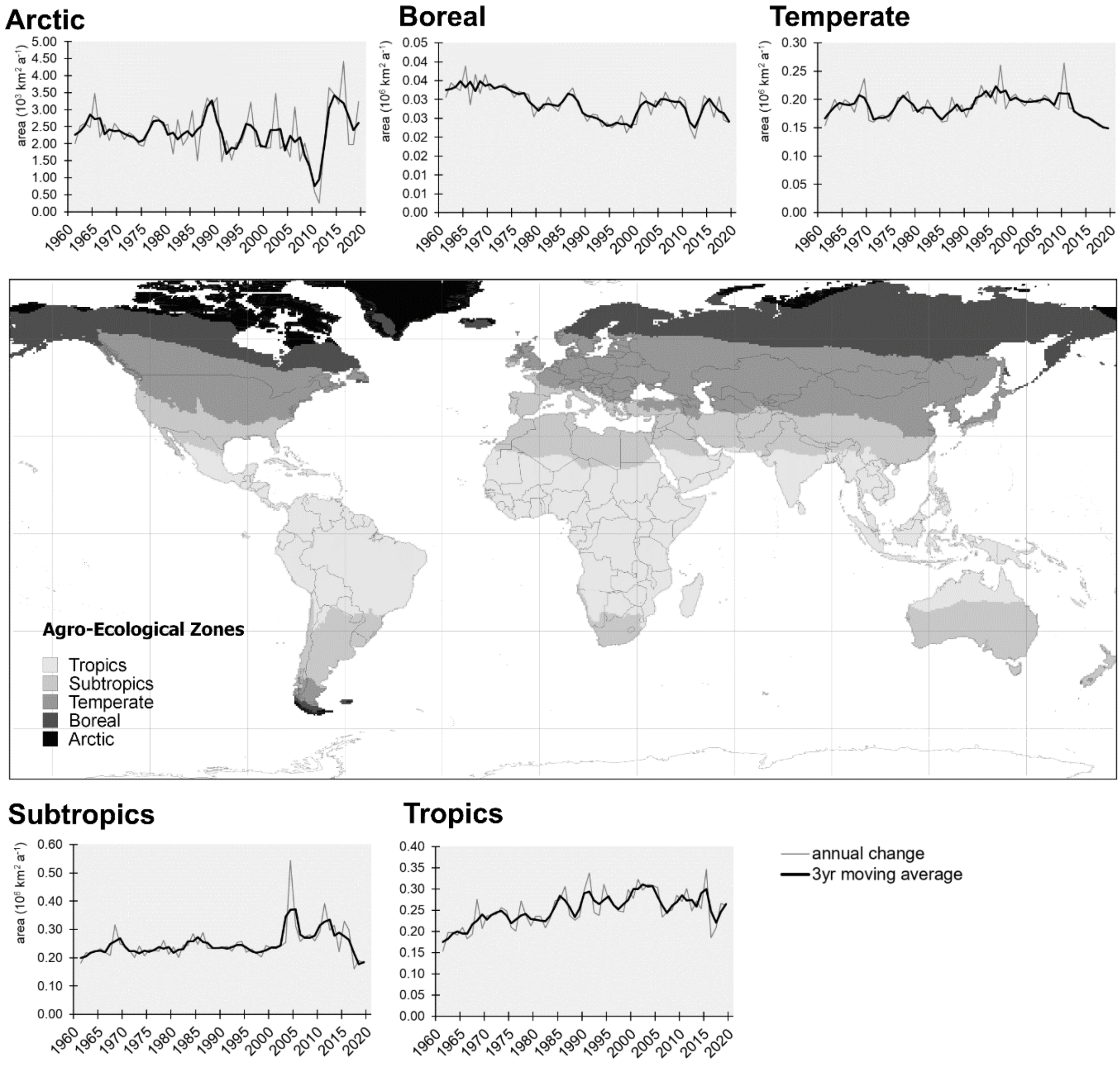

Supplementary Figure 3: Annual rate of land use change from HILDA+ between 1960 and 2019, aggregated by agro-ecological zones (retrieved from IIASA/FAO [26]).

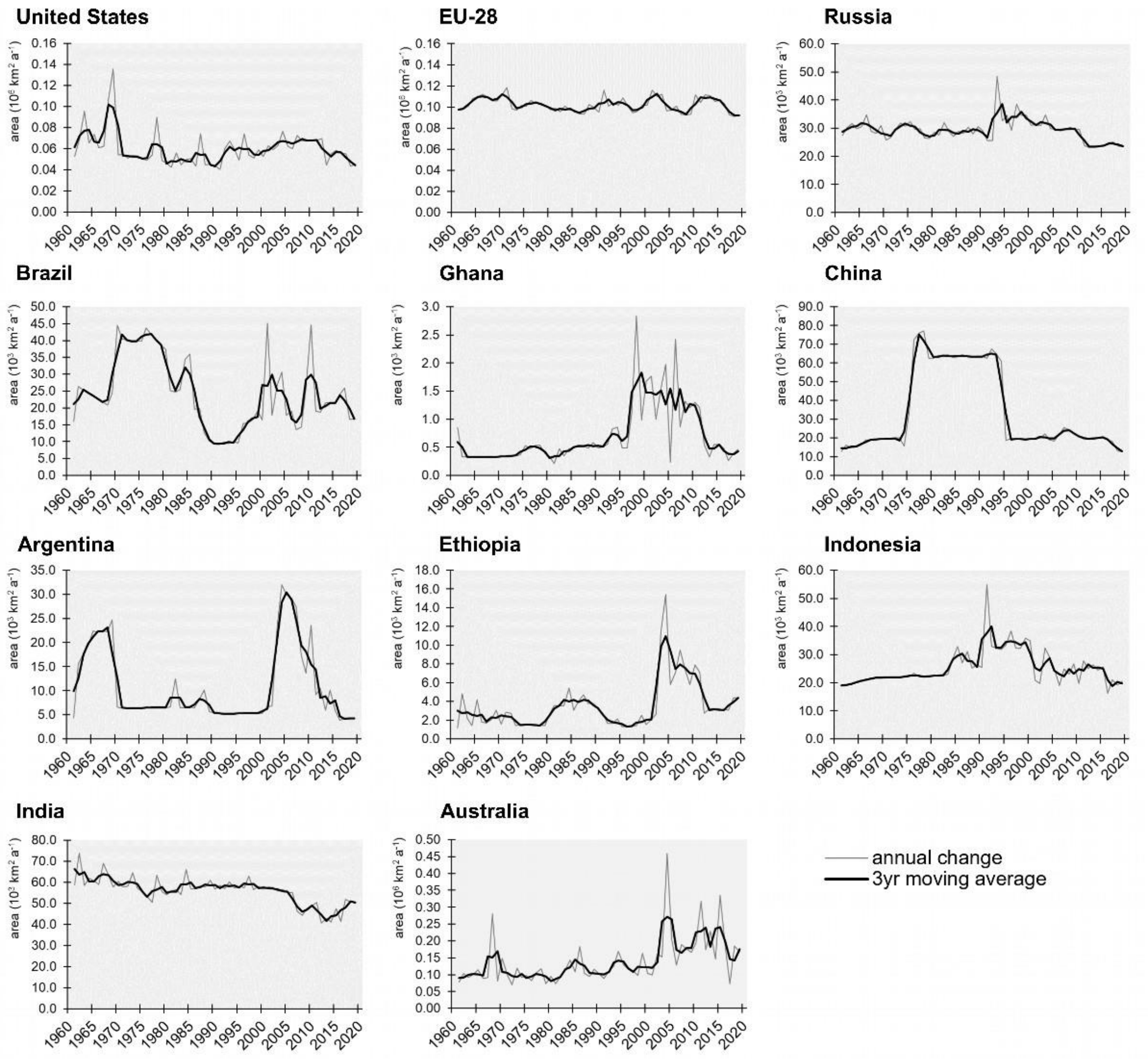

Supplementary Figure 4: Annual rate of land use change from HILDA+ between 1960 and 2019 for different countries and world regions.

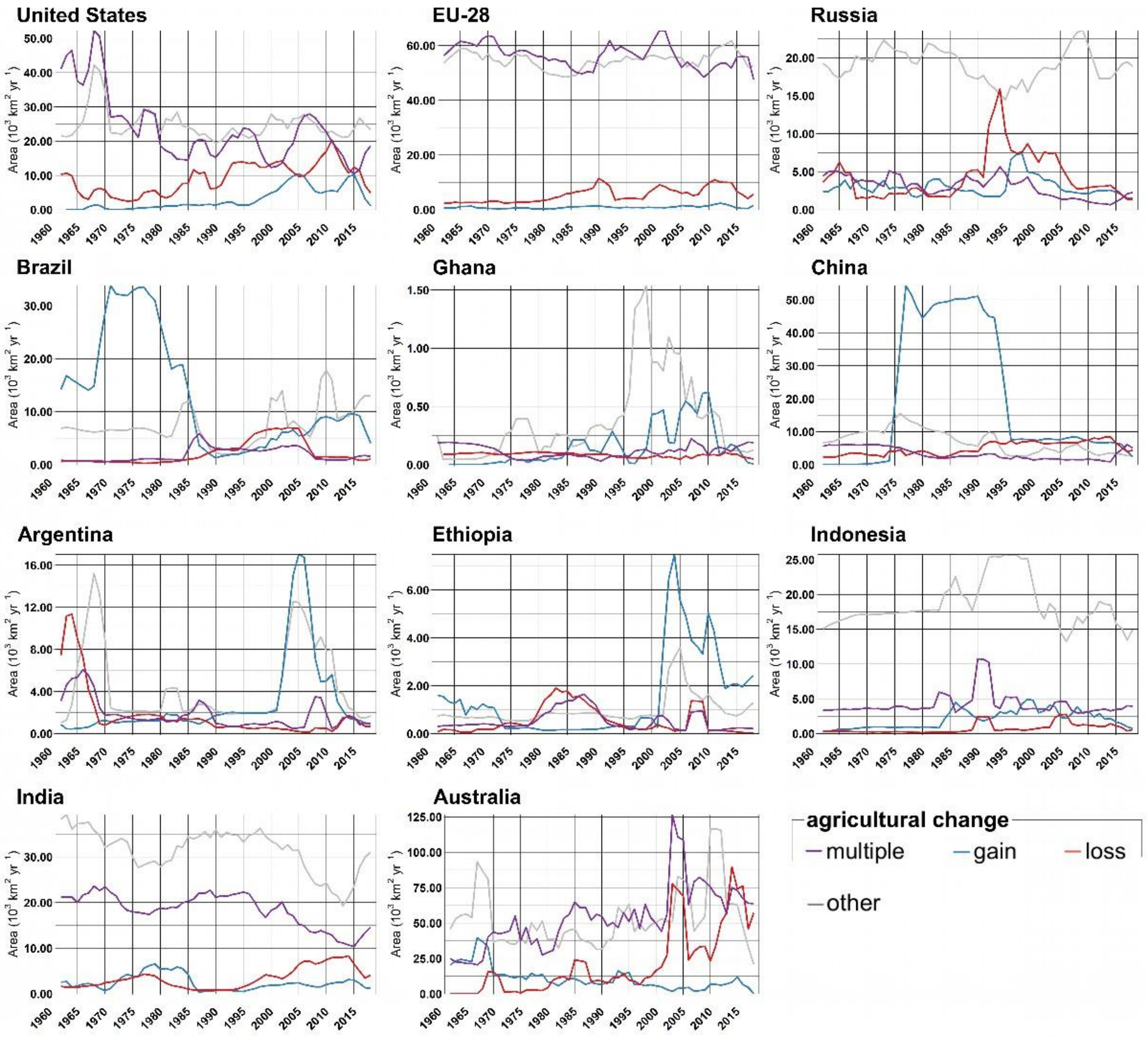

Supplementary Figure 5: Annual rates of land use change between 1960 and 2019 related to agriculture (gains, losses, multiple change events) from HILDA+ for different countries and world regions. Lines are smoothed using a 3-yearly mean.

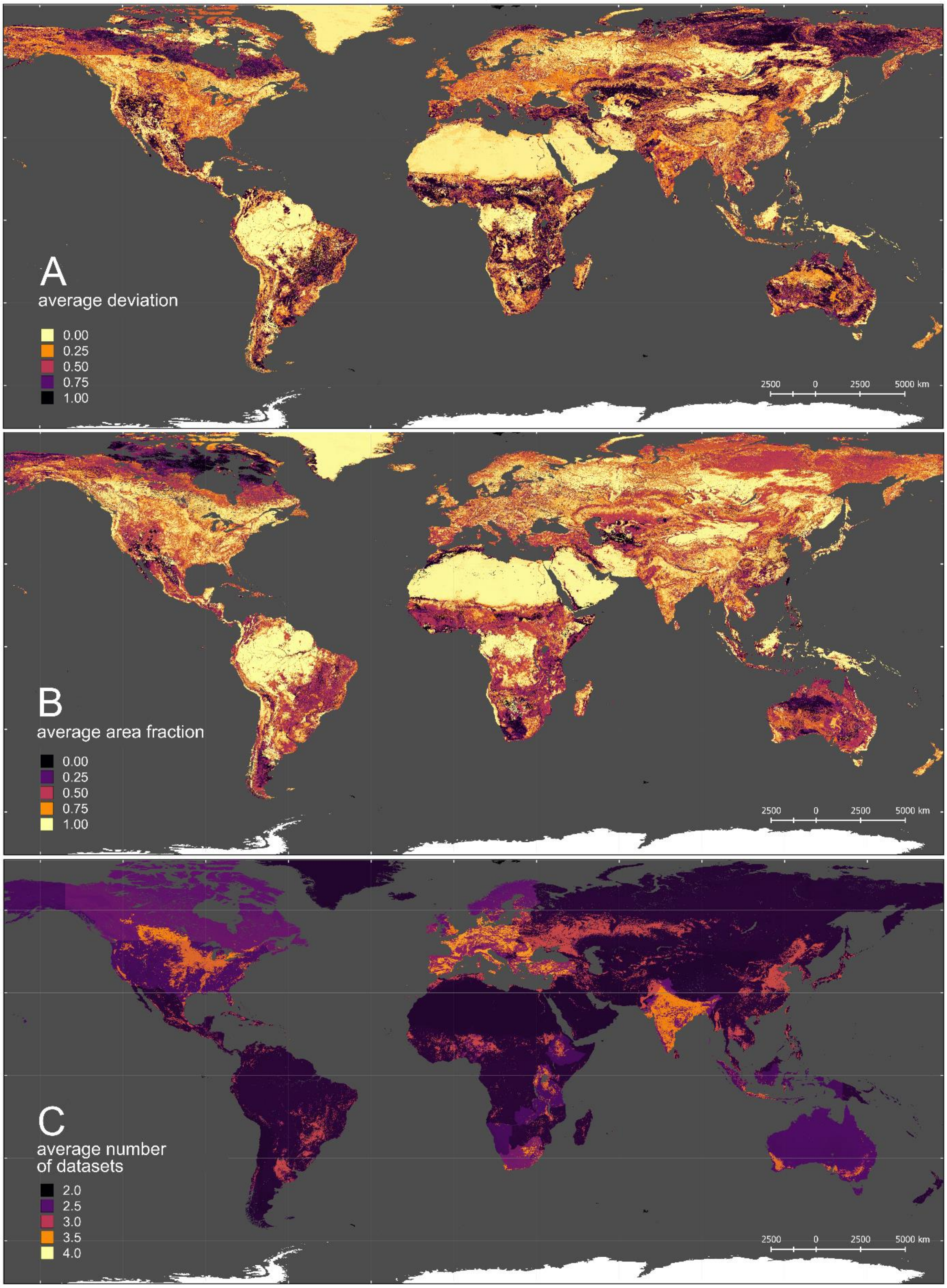

Supplementary Figure 6: Spatial distribution of global mean uncertainty information for HILDA+. Global land use/cover change 1960-2019: Multi-year mean of A) maximum deviation of class area fraction, B) LUC class area fraction, C) number of available datasets. Layers were derived on an annual basis for each indicated LUC

category by HILDA+ and averaged for the entire period (1960-2019). Note that, for LUC category 3: Pasture/rangeland, class area fractions refer to grassland and, for LUC category 5: Unmanaged grass /shrubland, class area fractions comprise grassland and shrubland.

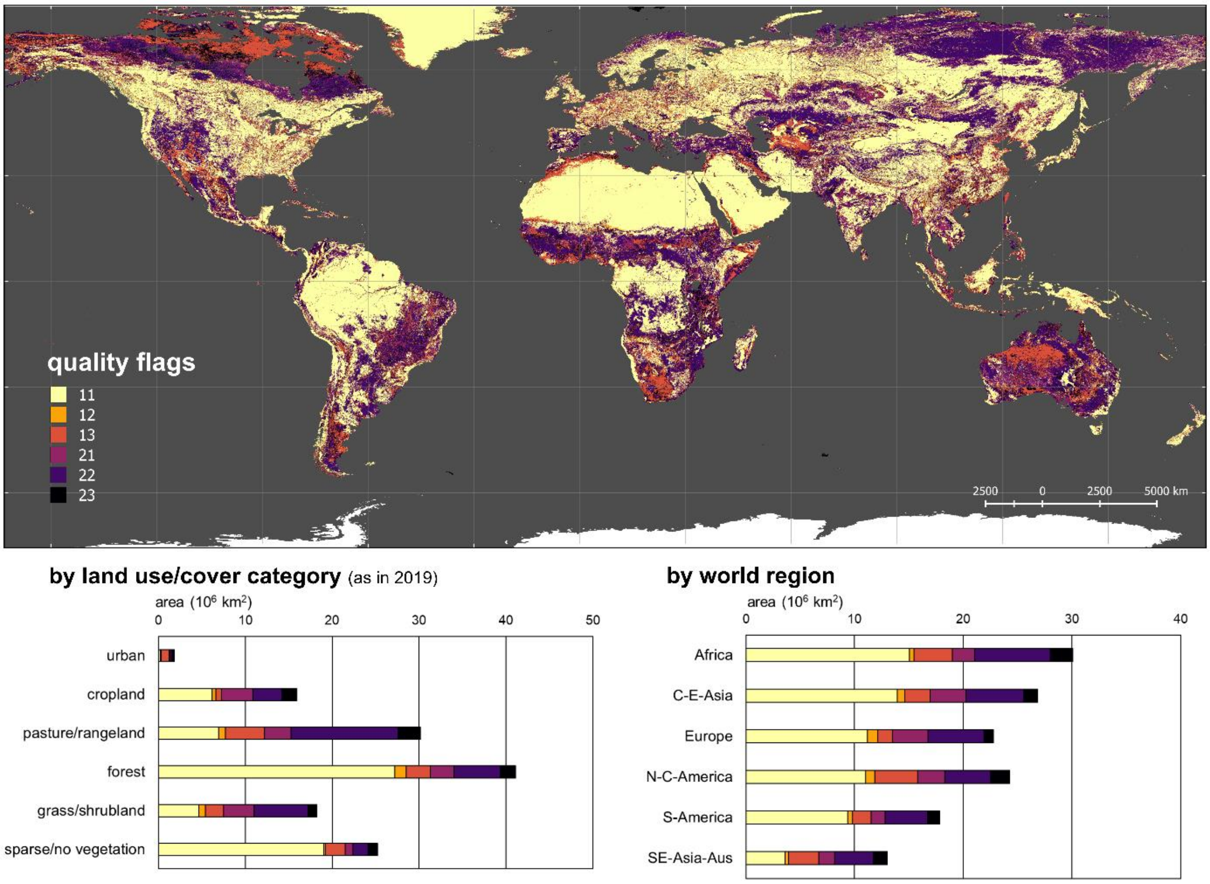

Supplementary Figure 7: Spatial distribution of multi-year mean quality flags for HILDA+ global land use/cover change 1960-2019. Quality flags: Good agreement with high (11), moderate (12) and low (13) class coverage; Low agreement with high (21), moderate (22) and low (23) class coverage.

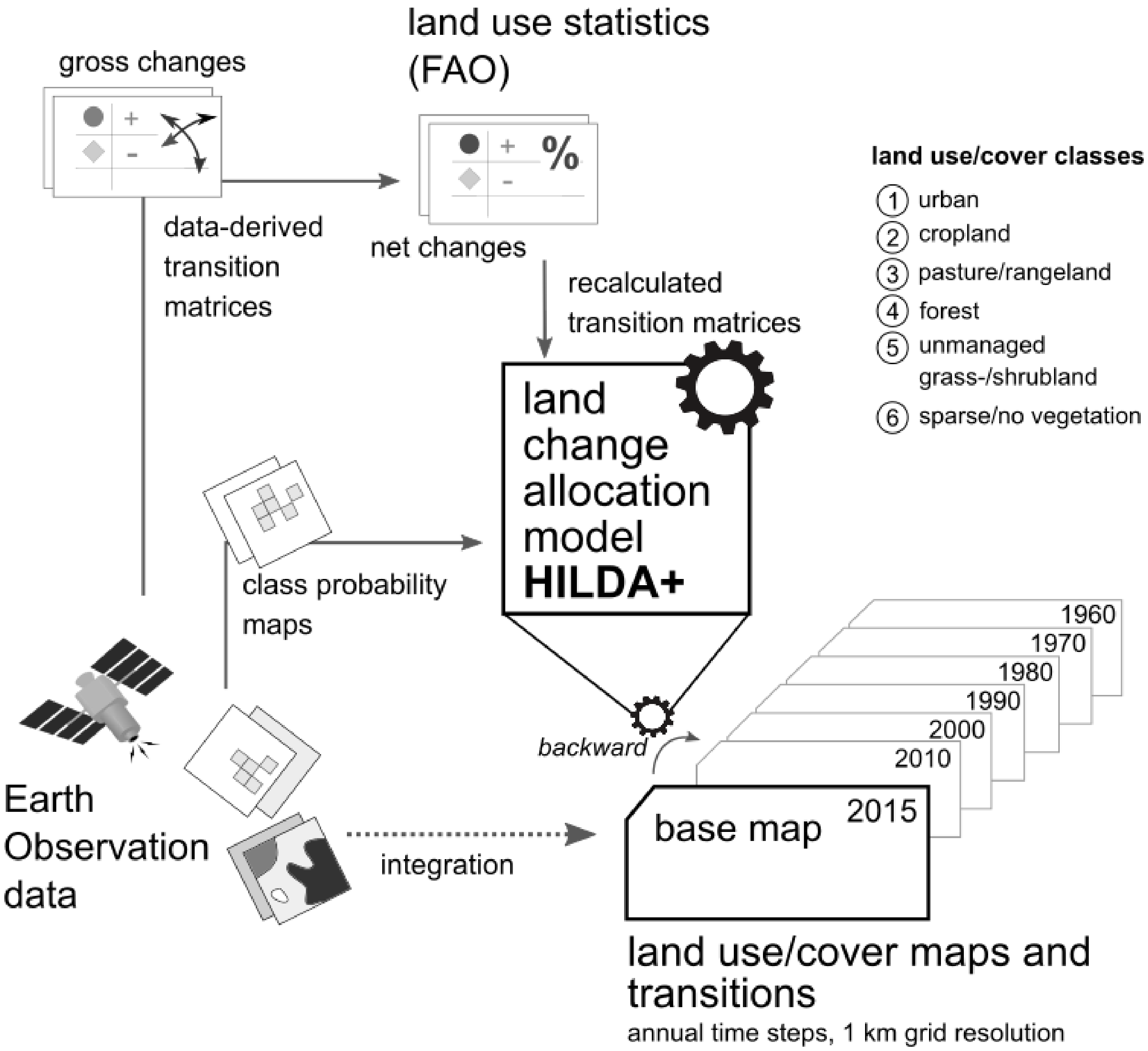

Supplementary Figure 8: Graphical overview of the HILDA+ (HIstoric Land Dynamics Assessment+) framework, a data-driven global land use/cover change allocation model.

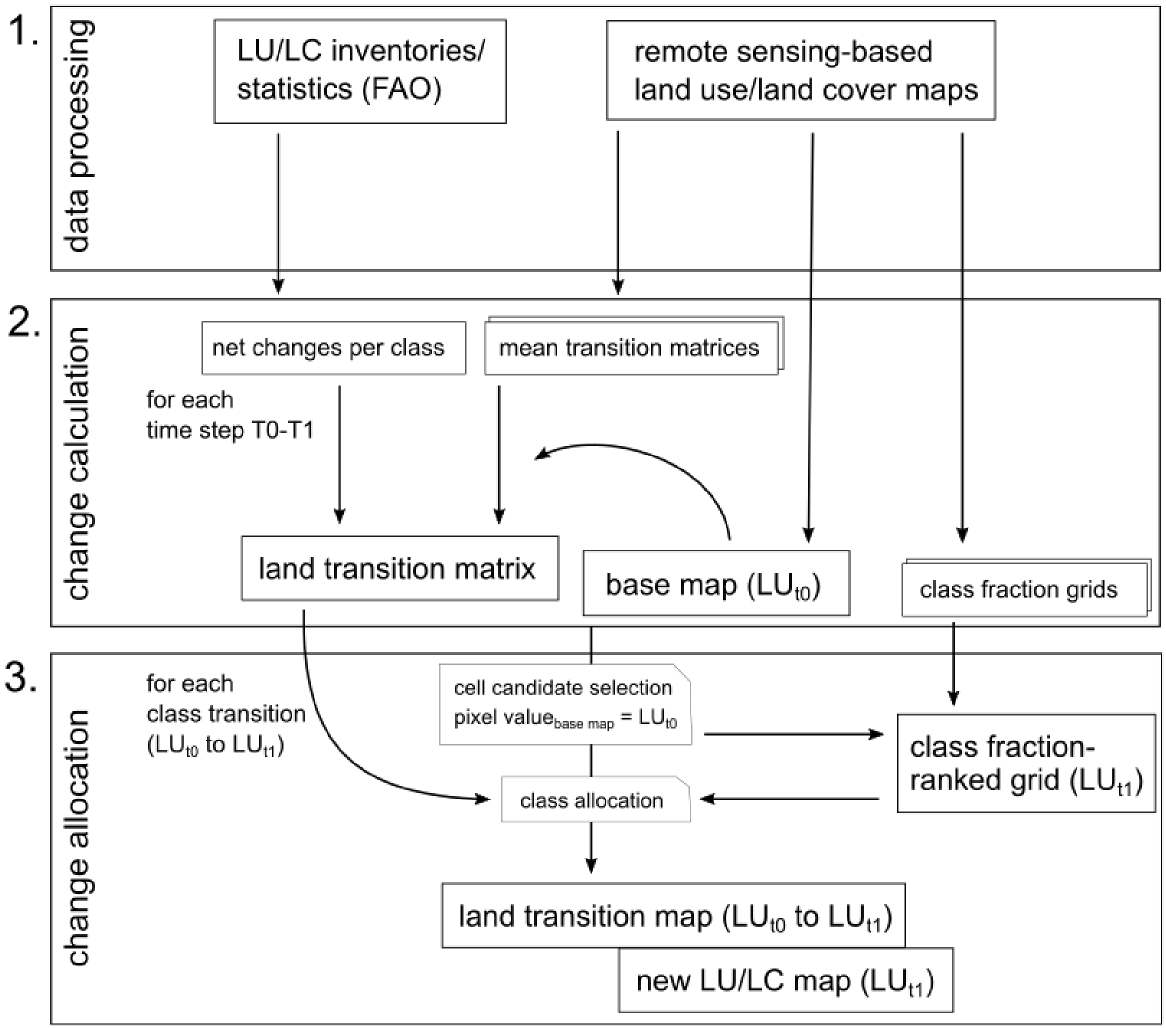

Supplementary Figure 9: Methodological steps of the land use/cover (LUC) change allocation procedure. (1) Input datasets are pre-processed and harmonised. (2) LUC change matrices are calculated for each time step (annually) and each country. In the backward mode (2015-1960), LU_{t0} refers to LUC classes in a specific year (time step 0), LU_{t1} refers to a LUC classes in the previous year (time step 1). In the forward mode (2015-2019), LU_{t1} refers to a LUC classes in the subsequent year (time step 1). (3) All combinations of land transitions between LUC classes are iteratively allocated on the map.

Supplementary Table 1: Land use/cover (LUC) datasets used for HiLDA+ LUC reconstruction and their specifications (thematic, spatial and temporal coverage).

Dataset and reference	Used thematic coverage	Spatial coverage	Used temporal coverage	Spatial resolution	Data type
Copernicus LC100 [1] https://land.copernicus.eu/global/products/lc	LCCS 22 classes	global	2015-2019	100 m	raster
ESA CCI Land Cover [2] http://maps.elie.ucl.ac.be/CCI/viewer/download.php	LCCS 22 classes	global	1992-2015	300 m	raster
GLAD UMD VCF [3] https://glad.umd.edu/dataset/long-term-global-land-change	tree canopy, bare ground, short vegetation	global	1982-2015	0.05 deg	raster
GLC2000 [4] http://forobs.jrc.ec.europa.eu/products/glc2000/glc2000.php	FAO LCCS 22 classes	global	2000	1 km	raster
GLCNMO [5] https://globalmaps.github.io/glcnmo.html	LCCS 22 classes	global	2003<br>2008, 2013	30 arc sec<br>15 arc sec	raster
Global Human Settlement Layer (GHSL) [6] https://ghslsys.jrc.ec.europa.eu/datasets.php	built-up area (fractional)	global	1975, 1990, 2000, 2014	1 km	raster
Global Urban Footprint (GUF) [7] https://www.dlr.de/eoc/en/desktopdefault.aspx/tabid-11725/20508_read-47944/	built-up area (fractional)	global	2011/12	2.8 arc sec	raster
GlobCover [8] http://due.esrin.esa.int/page_globcover.php	LCCS 22 classes	global	2005/2006, 2009	300 m	raster
Globeland30 [9] http://www.globeland30.org	10 LULC classes	global	2000, 2010	30 m	raster
Gridded Livestock World v3 (GLW) [10] http://www.fao.org/livestock-systems/en/	density of ruminants	global	2010	5 arc min	raster
Hansen GFC [11] https://earthenginepartners.appspot.com/science-2013-global-forest/download_v1.5.html	tree cover (fractional) loss and gain year	global	2000-2015	30 m	raster

MODIS MCD12Q1 [12] https://lpdaac.usgs.gov/products/mcd12q1v006/	IGBP 17 classes	global	2001-2013 (yearly)	500 m	raster
Ramankutty cropland (update from [13]) http://www.ramankuttylab.com/data.html	cropland	global	2000	5 arc min	raster
AAFC Land Use Canada [14] https://open.canada.ca/data/en/dataset/18e3ef1a-497c-40c6-8326-aac1a34a0dec	15 LULC classes	Canada	1990, 2000, 2010	30 m	raster
Australia DLCD V2.1 [15] https://ecat.ga.gov.au/geonetwork/srv/eng/catalog.search#/metadata/83868	LCCS 22 classes	Australia	2002-2014	500 m	raster
CORINE [16] https://land.copernicus.eu/pan-european/corine-land-cover/view	44 LULC classes with change layers	Europe (changing extent)	1990, 2000, 2006, 2012, 2018	100 m	raster
LULC classification of India [17] https://daac.ornl.gov/cgi-bin/dsviewer.pl?ds_id=1336	11 LULC classes (IGBP scheme)	India	1985, 1995, 2005	100 m	raster
MoEF Indonesia [18] http://webgis.dephut.go.id:8080/kemenhut/index.php/en/feature/download	22 LULC classes	Indonesia	2000, 2003, 2006, 2009	300 m	raster
NLCD Land Cover (CONUS) [19] https://www.mrlc.gov/data?f%5B0%5D=category%3Aland%20cover	16 LULC classes	U.S.	2001, 2006, 2011	30 m	raster
RCMRD Land Cover [20] http://opendata.rcmrd.org/search?tags=land%20cover	6 LULC classes with country-specific sub-classes	Botswana, Ethiopia, Lesotho. Malawi, Namibia, Rwanda, Tanzania, Uganda, Zambia	different years between 2000 and 2014	30 m	raster
South Africa Land Cover [21,22] http://www.sasdi.net/sresults.aspx?text=Land+cover&offset=0&f_text=land%20cover	35/72 LULC classes	South Africa	1990, 2013-14	30 m	raster

* LULC = land use/land cover, LCCS = Land Cover Classification System, IGBP = International Geosphere-Biosphere Programme

Supplementary Table 2: Defined target land use/cover categories of HILDA+

Final land use/cover category	Definition and description
1. Urban	Artificial surfaces, urban and built-up areas, including urban parks and sports areas, green spaces, industrial, deposits, extractions sites (mining etc.)
2. Cropland	Herbaceous and woody crops (also for hay production) including tree/shrub crops, orchards, plantations, multiple/layered crops, incl. mosaics (with cropland area fraction ≥ 40 %)
3. Pasture/Rangeland	Managed herbaceous plants (cover ≥ 10%) including managed grasslands (e.g. prairies, steppes, savannah, mosaics with tree/shrubs): grasslands or meadows used for e.g. livestock grazing or hay production with different intensities
4. Forest	Trees with > 5m height (cover ≥ 10 %) including forest plantation, trees on seasonally or permanently flooded areas, including mangroves
5. Unmanaged grass-/shrubland	Natural herbaceous plants (cover ≥ 10%) including grasslands (e.g. prairies, steppes, savannah, mosaics with tree/shrubs) or natural shrub cover (≥ 10 %), including permanently or regularly flooded areas (wetlands), (herbaceous) wetlands
6. Sparse/no vegetation	Bare areas, sparse vegetation (2-10 %), snow and ice, rocks, sand, mudflats

Supplementary Table 3: Generalised land cover classes used for dataset harmonisation

Land cover category	Copernicus LC100	ESA CCI LC	GLC2000	GLCNMO	GlobCover	Globeland30	MODIS MCD12Q1	Definition and description
1. Urban	50	190	22	18	190	80	13	Artificial surfaces, urban and built-up areas, including urban parks and sports areas, green spaces, industrial, deposits, extractions sites (mining etc.)
2. Cropland	40	10-12, 20, 30, 40	16-18	11-13	11, 14, 20, 30	10	12, 14	Herbaceous and woody crops (also for hay production) including tree/shrub crops, orchards, plantations, multiple/layered crops, incl. mosaics (with cropland area fraction ≥ 40 %)
3. Grassland	30	110, 130	13	8-9	120, 140	30	9, 10	(Natural) herbaceous plants (cover ≥ 10%) including grasslands: prairies, steppes, savannah, mosaics with tree/shrubs
4. Forest	111-116, 121-126	50, 60-62, 70-72, 80-82, 90, 100, 160, 170	10	1-5	40, 50, 60, 70, 90, 100	20, 27	1-5, 8	Trees with $>$ 5m height (cover ≥ 10 %) including forest plantation, trees on seasonally or permanently flooded areas, including mangroves
5. Shrubland	20, 90, 100	120-122, 140, 180	11-12	6-7, 10, 15	110, 130, 150, 170, 180	40, 50, 70	6-7, 11	Shrub cover (≥ 10 %), including permanently or regularly flooded areas (wetlands), (herbaceous) wetlands
6. Sparse/no vegetation	70	150-153, 200-202, 220	14, 19, 21	16-17, 19	200, 220	90, 100	15-16	Bare areas, sparse vegetation (2-10 %), snow and ice, rocks, sand, mudflats

Supplementary Table 4: Quality flags for HILDA+ Global land use/cover change 1960-2019: Rules are based on the global multi-year mean deviation and class area fraction of the respective LUC category from available datasets (see Supplementary Table 5).

Quality flag	Category name	Definition/rules
11	good agreement/<br>high class coverage	deviation ≤ 0.4/<br>class area fraction ≥ 0.6
12	good agreement/<br>moderate class coverage	deviation ≤ 0.4/<br>$0.4 >$ class area fraction < 0.6
13	good agreement/<br>low class coverage	deviation ≤ 0.4/<br>class area fraction ≤ 0.4
21	low agreement/<br>good class coverage	deviation > 0.4/<br>class area fraction ≥ 0.6
22	low agreement/<br>moderate class coverage	deviation > 0.4/<br>$0.4 >$ class area fraction < 0.6
23	low agreement/<br>low class coverage	deviation > 0.4/<br>class area fraction ≤ 0.4

Supplementary Table 5: Rules for assembling class probability maps for the target LUC categories

Land use/cover category	Rule for probability maps
1. Urban	Mean (all available year-specific urban area fractions)
2. Cropland	Mean (all available year-specific cropland area fractions)
3. Pasture/rangeland	Mean (Mean [all available year-specific grassland area fractions], GLW ruminant density)
4. Forest	Mean (all available year-specific forest/tree cover area fractions)
5. Unmanaged grass-/shrubland	Mean (Mean [all available year-specific shrubland area fractions], Mean [all available year-specific grassland area fractions])
6. Sparse/no vegetation	Mean (all available year-specific other land area fractions)

Supplementary Table 6: Rule-set for calibrating the base map to FAO land use statistics [23]

Land use category	IF<br>base map class > FAO land use area	base map class < FAO land use area
**Forest**<br>(FAO: Forest)	Forest cells with lowest forest area fractions (ranked) were converted to the non-forest category with highest area fraction.	Non-forest cells with highest woody area fractions (ranked mean of forest and shrubland area fractions) were converted to forest area (excluding woody area fraction below 5 %).
**Cropland**<br>(FAO: Arable land and Permanent cropland)	Cropland cells with lowest cropland area fractions (ranked) were converted to the non-cropland category with highest area fraction (excluding forest).	Non-cropland cells (forest excluded) with highest cropland area fractions were converted to cropland area (excluding cropland area fraction below 5 %).
**Pasture/rangeland**<br>(FAO: Permanent meadows and pastures)	Pasture cells with lowest pasture probability (ranked sum of grassland area fraction and pasture/rangeland probability mask 2015, see Table 4) were converted to the non-pasture category with highest area fraction (excluding forest and cropland).	Non-pasture cells (cropland and forest excluded) with highest pasture probability (ranked sum of grassland area fraction and pasture/rangeland probability mask 2015, see Table 4) were converted to pasture area (excluding pasture probability below 5 %).

Supplementary Table 7: Countries with changes in area in 1960-2015: Former/subsequent names and years of change (start of records from succeeding country according to FAO)

Former countries	Countries in 2015	Year of change
Sudan (former)	Sudan, South Sudan	2011
Serbia and Montenegro	Serbia, Montenegro	2006
Belgium-Luxembourg	Belgium, Luxembourg	2000
Czechoslovakia	Slovakia, Czechia	1993
Ethiopia PDR	Ethiopia, Eritrea	1993
USSR	Russian Federation, Ukraine, Belarus, Armenia, Azerbaijan, Estonia, Georgia, Kazakhstan, Kyrgyzstan, Latvia, Lithuania, Republic of Moldova, Tajikistan, Turkmenistan, Uzbekistan	1992
Yugoslav SFR	Croatia, The former Yugoslav Republic of Macedonia, Slovenia, Bosnia and Herzegovina, Serbia and Montenegro	1992
Pacific Islands Trust Territory	Marshall Islands, Micronesia (Federated States of), Northern Mariana Islands, Palau	1991
Saint Christopher-Nevis-Anguilla	Anguilla, Saint Kitts and Nevis	1980
Leeward Islands	Antigua and Barbuda, Saint Christopher-Nevis-Anguilla, Montserrat, British Virgin Islands, Dominica	1961

References

1. Buchhorn, M. *et al.* Copernicus Global Land Service: Land Cover 100m: Epoch 2015: Globe. *Version V2 02* (2019).

2. ESA. *Land Cover CCI Product User Guide Version 2.* maps.elie.ucl.ac.be/CCI/viewer/download/ESACCI-LC-Ph2-PUGv2_2.0.pdf (2017).

3. Hansen, M. & Song, X.-P. Vegetation Continuous Fields (VCF) Yearly Global 0.05 Deg. (2018) doi:10.5067/MEASURES/VCF/VCF5KYR.001.

4. Bartholomé, E. & Belward, A. S. GLC2000: a new approach to global land cover mapping from Earth observation data. *Int. J. Remote Sens.* **26**, 1959–1977 (2005).

5. Tateishi, R. *et al.* Production of global land cover data – GLCNMO. *Int. J. Digit. Earth* **4**, 22–49 (2011).

6. Corbane, C., Florczyk, A., Pesaresi, M., Politis, P. & Syrris, V. GHS built-up grid, derived from Landsat, multitemporal (1975-1990-2000-2014), R2018A. *Eur. Comm. Jt. Res. Cent. JRC Doi* **10**, (2018).

7. Esch, T. *et al.* Breaking new ground in mapping human settlements from space – The Global Urban Footprint. *ISPRS J. Photogramm. Remote Sens.* **134**, 30–42 (2017).

8. Bontemps, S. *et al.* GLOBCOVER 2009-Products description and validation report. *URL HttpdueesrinesaintfilesGLOBCOVER2009ValidationReport22pdf* **2**, (2011).

9. Jun, C., Ban, Y. & Li, S. China: Open access to Earth land-cover map. *Nature* **514**, 434 (2014).

10. Gilbert, M. *et al.* Global distribution data for cattle, buffaloes, horses, sheep, goats, pigs, chickens and ducks in 2010. *Sci. Data* **5**, 180227 (2018).

11. Hansen, M. C. *et al.* High-Resolution Global Maps of 21st-Century Forest Cover Change. *Science* **342**, 850–853 (2013).

12. Friedl, M. A. & Sulla-Menashe, D. MCD12Q1 MODIS/Terra+Aqua Land Cover Type Yearly L3 Global 500m SIN Grid V006. (2019).

13. Ramankutty, N. & Foley, J. A. Estimating historical changes in global land cover: Croplands from 1700 to 1992. *Glob. Biogeochem. Cycles* **13**, 997–1027 (1999).

14. Agriculture and Agri-Food Canada (AAFC). Land Use 1990, 2000 & 2010, Dataset. https://open.canada.ca/data/en/dataset/18e3ef1a-497c-40c6-8326-aac1a34a0dec (2015).

15. Lymburner, L., Tan, P., McIntyre, A., Thankappan, M. & Sixsmith, J. Dynamic land cover dataset version 2.1. *Geosci. Aust. Canberra* (2015).

16. Feranec, J., Soukup, T., Hazeu, G. & Jaffrain, G. *European landscape dynamics: CORINE land cover data.* (CRC Press, 2016).

17. Meiyappan, P. *et al.* Dynamics and determinants of land change in India: integrating satellite data with village socioeconomics. *Reg. Environ. Change* **17**, 753–766 (2017).

18. Ministry of Enviornment and Forestry Republic of Indonesia (MoEF). Penupan Lahan, Kementerian Lingkungan Hidup dan Kehutanan. http://webgis.menlhk.go.id:8080/pl/pl.htm (2019).

19. Homer, C. *et al.* Completion of the 2011 National Land Cover Database for the conterminous United States–representing a decade of land cover change information. *Photogramm. Eng. Remote Sens.* **81**, 345–354 (2015).

20. Africa RCMRD-SERVIR. Land cover mapping for greenhouse gas inventories development project in east and southern Africa region. *Ethiop. Mapp. Agency Addis Ababa Ethiop.* (2015).

21. Thompson, M. South African Land Cover 1990 [Data set]. Department of Environment, Forestry and Fisheries. https://doi.org/10.15493/DEA.CARBON.10000051 (2019).

22. Department Of Environmental Affairs (DEA). 2013-14 SA National Land-Cover – broad parent classes [Data set]. Department of Environmental Affairs (DEA). https://doi.org/10.15493/DEA.CARBON.10000015 (2015).

23. Food and Agriculture Organization of the United Nations (FAO). FAOSTAT Land Use [Dataset], http://www.fao.org/faostat/en/#data/RL. (2019).

24. Food and Agriculture Organization of the United Nations (FAO). FAOSTAT Trade, Crops and livestock products [Dataset], http://www.fao.org/faostat/en/#data/TP. (2020).

25. Food and Agriculture Organization of the United Nations (FAO). FAOSTAT Production, Crops [Dataset], http://www.fao.org/faostat/en/#data/QC. (2020).

26. Fischer, G., van Velthuizen, H. T. & Nachtergaele, F. O. Global Agro-Ecological Zones Assessment: Methodology and Results. http://pure.iiasa.ac.at/id/eprint/6182/ (2000).
